# COVID‐19 and the UK water sector: Exploring organizational responses through a resilience framework

**DOI:** 10.1111/wej.12737

**Published:** 2021-06-07

**Authors:** Elizabeth Lawson, Sarah Bunney, Sarah Cotterill, Raziyeh Farmani, Peter Melville‐Shreeve, David Butler

**Affiliations:** ^1^ Centre for Water Systems University of Exeter Exeter UK; ^2^ Department of Mathematics Imperial College London London UK; ^3^ School of Civil Engineering University College Dublin Dublin Ireland

**Keywords:** COVID‐19, organisational response, resilience, Safe and SuRe, water sector

## Abstract

The unprecedented scale and impact of the COVID‐19 pandemic have required organizations to adapt all facets of their operations. The impact on the UK water sector extends beyond engineering and treatment processes, with social, economic and environmental consequences. Semi‐structured interviews were conducted with executives from 10 UK water companies to investigate the organizational response to the pandemic, and how their response impacted operational delivery. The Safe and SuRe framework was used to structure interview questions and analysis. Emergent themes of changes to customer behaviour, changes to operational practices and industry collaboration were mapped onto the framework and a ripple effect map developed. Lessons learnt highlight a failure to adequately prepare for the scale of the threat, the success of sector‐level collaboration and a need to embrace new ways of working.

## 1 INTRODUCTION

The COVID‐19 pandemic continues to alter the way we live and work, with the need to ensure safe and reliable water and wastewater services becoming more critical than ever because of the pivotal role hygiene plays in mitigating the spread of the disease (Poch et al., 2020). Governments worldwide enacted restrictions on national and international movements, to stop the spread of COVID‐19 (Nghiem et al., 2020). As the pandemic has continued, organizations have been required to creatively use existing resources, structures and processes and to develop alternative solutions to problems arising from the event.

In the United Kingdom, national restrictions were initially imposed on Monday (23 March 2020) and began easing on Saturday (4 July 2020), with residents only able to leave their homes to travel to work where necessary, to shop for essential items, to exercise once a day or to access medical care (Iacobucci, 2020). A number of sectors and professions, including the water industry, were identified as essential services with frontline staff defined as ‘key workers’. As highlighted by Farquharson et al. (2020), the resilience of the UK economy and wider society to the COVID‐19 pandemic largely depends on the ability of key workers and organizations to respond to and adapt to maintain the performance of key services (Cotterill et al., 2020).

Efforts to track the level of community infection of SARS‐CoV‐2 through the analysis of wastewater (Mao et al., 2020) and a focus on the efficacy of drinking water treatment processes (Maal‐Bared et al., 2020) have promptly been investigated by researchers across the globe. However, the pandemic's impact on water systems goes beyond engineering and treatment processes with social, economic and environmental consequences, such as increases in demand, reductions in revenue and an increase in public interest in local water environments, already occurring.

The ability to meet organizational resilience objectives will be a key challenge for the UK water sector going forward. Organizational resilience is a process, where organizations actively review their operational procedures and response to both anticipated and unanticipated threats and hazards (Bruijne et al., 2010; Weick et al., 1999). The success of this process lies in an organizations capacity to mitigate, adapt, cope and learn from a crisis (Butler et al., 2016; Weick & Sutcliffe, 2001).

For many, the COVID‐19 pandemic has tested this process and the ability of organizations to anticipate the impact and consequences within a complex socio‐technical environment. A multi‐agency approach to secure resilient supply chains in preparation for Brexit helped ensure that there were adequate plans in place at the start of the pandemic (Cotterill et al., 2020). Many UK water companies also perceived that they had adequate business continuity plans and contingency arrangements in place. However, it is unknown how resilient these organizations will be to an event of prolonged duration (Cotterill et al., 2020). How water sector organizations respond to the current threat posed by COVID‐19 will not only highlight, but also impact, the reliability, resilience and sustainability of the sector going forward.

This study aims to analyse the organisational response of the UK water sector to the COVID‐19 pandemic using the Safe and SuRe framework and assess how the initial response impacted operational delivery during the first wave of the pandemic.

### 1.1 The Safe and SuRe intervention framework

**FIGURE 1:**
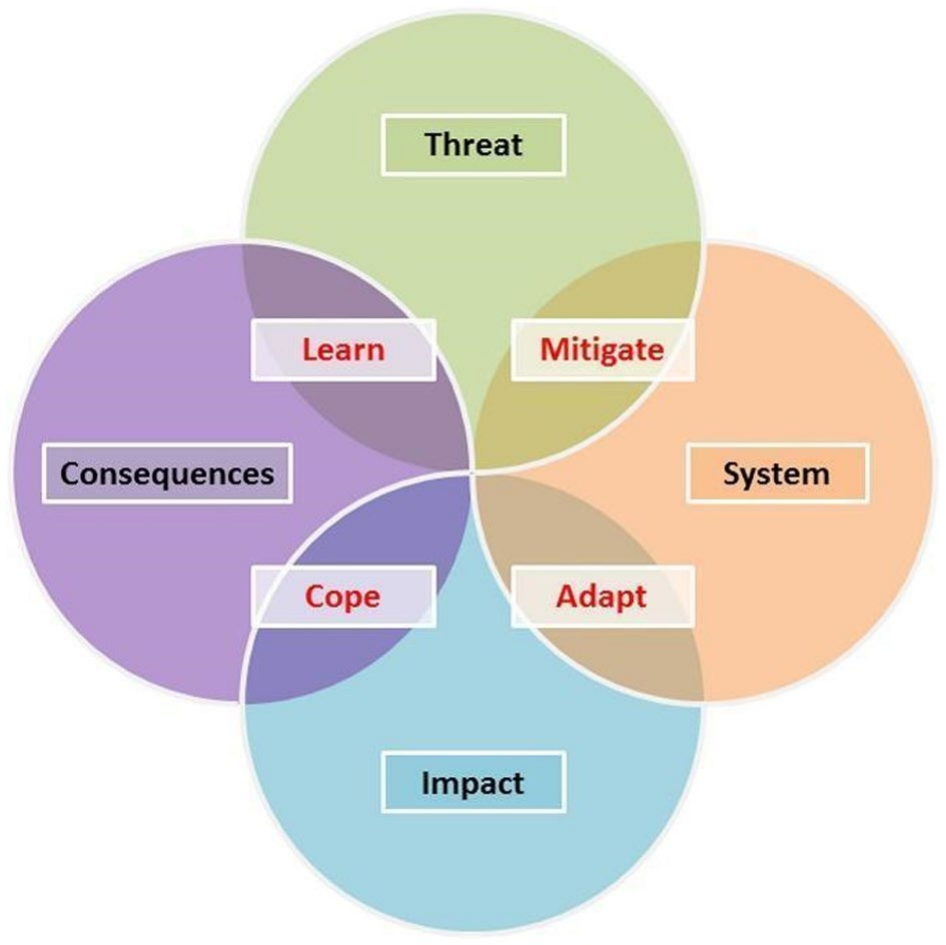
Safe and SuRe framework (Butler et al., 2016)

The Safe and SuRe approach (Butler et al., 2014, 2016) is based on the premise that urban water systems have traditionally been designed to provide a reliable (Safe) service. However, in an era of unknown and emerging threats, new approaches are required to enable the evolution or transition to resilient and sustainable systems. The Safe and SuRe intervention framework (Figure 1) is a theoretical framework that provides a representation of the relationship between threats and their consequences (Butler et al., 2016) and enables opportunities to identify interventions that would increase system resilience. Resilience is defined as ‘the degree to which the system minimizes level of service failure magnitude and duration over its design life when subject to exceptional conditions’ (Butler et al., 2014), with threats, system failure modes, impacts and consequences defined in Table 1. The framework clarifies the role of the water system in ‘mediating between threats and compliance with defined levels of service (impacts)’ (Butler et al., 2016, p. 68).

**TABLE 1:** Safe and SuRe terminology and definitions

	Definition
Mitigation	Any physical or non‐physical action taken to reduce the frequency, magnitude or duration of a threat
Adaptation	Action taken to modify specific properties of the water system to enhance its capability to maintain levels of service under varying conditions
Coping	Any preparation or action taken to reduce the frequency, magnitude or duration of an impact on a recipient
Learning	Embedding experiences and new knowledge in best practice
Threat	Any event that has the potential to affect system performance
System failure mode	How the system fails as a result of the threat
Impact	Degree of non‐compliance with a defined level of service
Consequence	Any outcome because of the effects of non‐compliance with a defined level of service

Mitigation, adaptation, coping and learning, which are defined in Table 1, are processes, procedures or actions (interventions) that can be designed or implemented at different interacting levels of the system in question. Together the implementation of the identified interventions aims to minimize the frequency, magnitude and duration of the consequences of threats to urban water management systems (Bryan, 2017). The framework also provides a logical foundation for the analysis of system reliability, resilience and sustainability through consistency in assessment methodologies and methodical identification of modes of intervention (Baker et al., 2018).

Application of the framework for analysis in this research aims to assess the performance of the UK water sector when subject to the COVID‐19 pandemic. This research applied the Safe and SuRe framework for analysis using a ‘top‐down’ or threat‐based approach (Butler et al., 2014) to the socio‐technical system of the UK water sector. The definitions used throughout the analysis are those outlined and applied in the Safe and SuRe framework (Table 1; Butler et al., 2014, 2016).

## 2 METHOD

### 2.1 Interview design

Semi‐structured interview questions were designed as a follow up to an online survey on COVID‐19 and the UK water sector (Cotterill et al., 2020). The questions were structured around the four phases of intervention (mitigation, adaptation, coping and learning) Butler et al. (2014). The questions explored: pandemic preparations, adaptations post‐pandemic emergence, other large‐scale threats during the period that required further intervention, unanticipated challenges, effectiveness of coping mechanisms, reflections on measures taken; and lessons learnt. The questions were tested in advance by the wider research team and members of industry to ensure clarity and suitability.

### 2.2 Data collection and analysis

A total of 11 semi‐structured interviews were conducted with industry executives from UK‐based water companies, with one company providing two participants to be included in the study. Interviews took place between 21 July 2020 and 13 November 2020. The sampling strategy used, targeted senior‐level individuals who were directly involved in the management of their organization's COVID‐19 operational response. Participants took part and commented on behalf of their organizations. Fifteen initial invitations to participate in the research were sent out by the Chartered Institution of Water and Environmental Management (CIWEM) who helped facilitate the research.

The interviews were conducted via Microsoft Teams and were audio‐recorded and transcribed. Both transcription and coding were conducted using a qualitative data analysis software package NVivo (NVivo v.12, QSR International). All transcripts were read through repeatedly, to allow familiarization with the information, and coded into themes. The coding process was conducted independently for each interview by two researchers, and later discussed and found to be similar thus providing validation.

**FIGURE 2:**
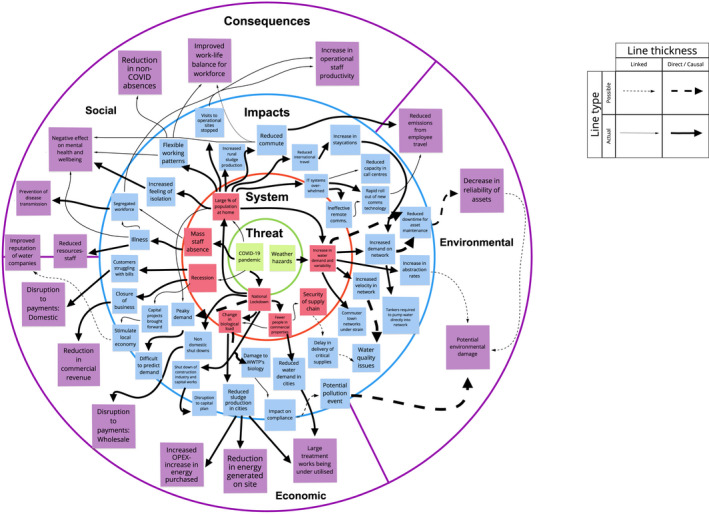
Ripple effect map

**FIGURE 3:**
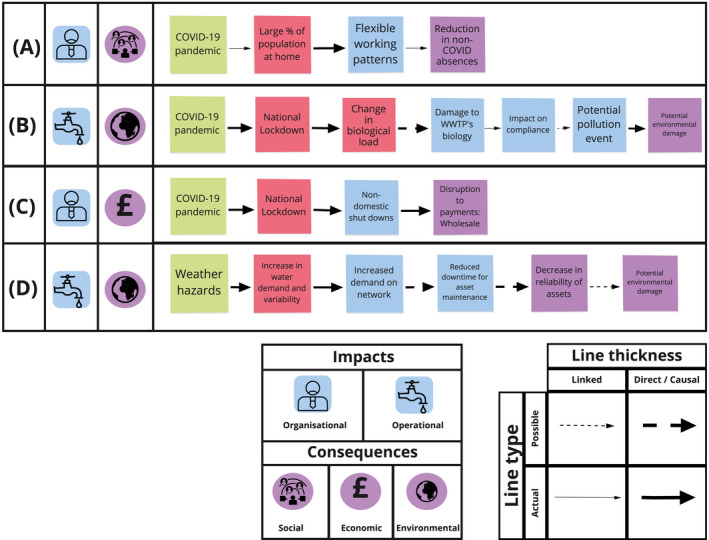
Example routes through REM

The second level of analysis involved understanding the context of the information with regards to the Safe and SuRe framework. Emergent themes were grouped into the four intervention categories of mitigation, adaptation, coping and learning and plotted onto the Safe and SuRe framework. Threats, system failure modes, impacts and consequences that were discussed within the interviews were also mapped onto the framework. Ripple effect mapping (REM), a qualitative method for conducting impact evaluation using a diagramming process that represents connections hierarchically (Kollock et al., 2012), was then conducted for threats, system failure modes, impacts and consequences (Figure 2). This provided a visual representation of the implications of the threat. Both actualized and potential system failure modes, impacts and consequences that were discussed in the interviews are included in the REM as, at the time of writing, the pandemic remains an ongoing incident. The second level of analysis and creation of the REM was conducted by three researchers collaboratively and further validated by the wider research team. Although validation of the REM by interview participants would have been preferable, this may have compromised the anonymity of interview participants, and their willingness to discuss incidents openly, and was therefore not pursued. Four example routes through the REM are shown in Figure 3.

## 3 RESULTS

### 3.1 Ripple effect map

The use of arrows in the REM demonstrates the direct causal and possible links between threat, system, impact and consequence (Figure 2). The COVID‐19 pandemic was identified as a ‘threat multiplier’ (Neal, 2020). The interaction with the ongoing weather event of reduced rainfall and higher than average temperatures increased the complexity and cascading nature of the system failure modes, their impacts and subsequent consequences.

During April and May 2020, the United Kingdom saw higher than average temperatures with prolonged periods of reduced rainfall (Madge, 2020; Met Office, 2020), coinciding with the height of the first wave of the pandemic and national lockdown. As a result, water companies across the United Kingdom reported higher than ever levels of demand (Water Briefing, 2020), with changes to peak demand and distribution patterns seen across the United Kingdom (Table 2, R1; Figure 3d). Although the nationwide lockdown resulted in large‐scale commercial and industrial closures, the reduction in demand from the non‐domestic sector failed to cover the overall increase (Table 2, R2). Such closures seen across the non‐domestic sector resulted in an economic consequence through disruption to wholesale payments (Figure 3c).

**TABLE 2:** Increase in water demand

Ref	Measure	Qualitative explanation
R1	Water demand	‘Because lockdown coincided with dry weather [people were] out in their gardens because there was nowhere else to go’ ‘We saw demand increase by 350 million litres of water a day over a 36 hour period, which is huge’ ‘I saw really high demand in areas where I’ve never seen really high demand before’
R2	Domestic vs commercial water demand	‘while commercial demand dropped off completely, water demand from domestic customers increased to more than cover … that commercial drop off’

The Safe and SuRe framework focuses on the middle‐based analysis (middle states), which recognizes that it is impossible to identify every threat to a system, and instead focuses on failure modes of systems and their related impacts (Butler et al., 2016). Middle states occur as a result of threats and represent all potential modes of failure for a given system. One benefit of this approach is that multiple threats that result in the same failure mode can be addressed with a single analysis, enabling a more comprehensive resilience assessment (Butler et al., 2016). It is however important to note that while multiple threats can result in the same middle state, there are still multiple different ways in which a system can fail (Butler et al., 2016). Middle states can be further classified as either internal or external to the system and functional (operational) or structural. The COVID‐19 pandemic directly contributed to modes of failure, including but not limited to, the national lockdown, the closure of commercial entities and large‐scale industries. There were also examples of secondary failure modes, such as a change in biological load (Figure 3b), which were not a direct result of COVID‐19 but a result of the national lockdown.

The cascading effect of related impacts not only required additional coping mechanisms to be implemented but also resulted in social, economic and environmental consequences (Figures 2 and 3). The interventions put in place to mitigate, adapt, cope and learn from the subsequent failure modes, impacts and consequences are further outlined in the next section. The relative success or effect each intervention had, dictated which impacts and consequences each organization witnessed.

Figure 2 shows how cascading effects occur within a single tier or ripple, as the effects of one failure mode result in failures in other areas of the system, highlighting the complexity and interconnectedness that exists within such organizations. Resulting cascading impacts can also be seen as the effects of system failure modes (that lacked appropriate adaptations), propagate through the system, ending in yet further social, economic and environmental consequences. The effect of organizational and operational impacts and their resulting social, economic and environmental consequences is shown in four examples from the REM in Figure 3. Both Figures 2 and 3 highlight the complexity of the system and its corresponding failure modes and impacts which were further exacerbated by simultaneous threats.

### 3.2 Mitigation

Participants discussed mitigation measures that were taken to reduce the frequency, magnitude and duration of COVID‐19 impacts on organizational performance and operational delivery (Table 3). Referring to the organizational level, discussion around mitigation measures was concentrated on the existence of pandemic contingency and business continuity plans. Although all participants referred to the availability of contingency plans, many described the ‘dusting down’ of plans in order to more adequately address the threat they were facing (Table 3, M1).

**TABLE 3:** Mitigation measures implemented by organizations

Ref	Measure	Qualitative explanation
M1	Pandemic contingency plans	‘Yeah we had a pandemic contingency plan’ ‘I think it is fair to say that the plans probably covered 60% of what we experienced so it was a very quick rehash of the plans to try to understand what we needed’ ‘While we have had BSE, bird flu, SARS and Swine flu … and had business continuity plans for all of them, [they were] nothing on the scale of this’
M2	Past incident management experience	‘The water industry is used to dealing with incidents so … it was not that big of a deal, we just flicked into incident mode and managed it’
M3	Brexit preparations	‘I think the industry itself had quite a collective response towards Brexit … so we just kicked back in and carried on the Brexit preparations that we had been doing, so that worked really well’
M4	Additional staff training or ‘upskilling’	‘We started to look at how many people we could train to carry out frontline critical roles in case our normal operators were unable to work due to COVID. And we trained over I think about a 2‐month period about 300 staff who are in non‐frontline roles to take on frontline roles if that would be needed …. Yeah preparations started quite early for that work about January time I would say’
M5	Changes to operational working practices	‘People were not allowed in the control rooms they would have to do remote handover they would have to keep separation between the maintenance and ops [operations] teams so they would not mingle … we did start to put in measures to stop people physically interacting as much as they would have done otherwise’.
M6	Preparation for working from home	‘We were discussing whether we should do a mass work from home exercise as we agreed on the Monday [2 March] morning myself and a couple of directors went to see the CEO and said look we are going to do this working from home exercise. It is going to be quite disruptive but you know on the horizon we can see lockdown coming’
M7	Customer support	‘So we have got about 3000 customers on our customer care register … we would keep them informed of anything going on in their area … and we do a delivery of bottled water for over the winter months ….So we did that again proactively at the beginning of April … we made the proactive decision to deliver bottled water to those customers upfront’
M8	Water network pressure	‘We sent out our fleet of 30‐odd tankers to pressurize the network in certain areas. [We] kept supplies going … under COVID restrictions … and customers did not know’

Failure to account for the scale of this event, and the associated impacts and consequences, was another theme presented in the analysis of the data. Although participants discussed the sector's history with regards to the need to prepare for and respond to previous global events, including infectious disease outbreaks, the scale of the COVID‐19 pandemic is not something organizations were prepared for (Table 3, M1). Past industry experience in dealing with, and learning from, both smaller routine and other large‐scale events were also referred to as actions that aided an organization's ability to implement measures to mitigate the threat they were now facing (Table 3, M2).

All participants spoke of the merits of the collaborative planning for the UK’s departure from the European Union that had been carried out at the industry scale. A platinum‐level group coordinated by Water UK focused on maintaining the supply of chemicals and other vital resources. As such plans already existed, they were easily implemented to mitigate any issues that could arise from the pandemic with regards to the security of supply (Table 3, M3).

Other mitigation measures taken by organizations included preparations taken for mass absenteeism, including training or ‘upskilling’ staff to operate treatment works, recruitment of university students to work in laboratories and contacting recently retired operational staff to assess their willingness to return to work if required (Table 3, M4). Participants also referred to operational interventions implemented in the weeks prior to the government mandated national lockdown. Such actions included isolating operational teams with specific specialist skills and minimizing contact between different teams working on operational sites and in laboratories, with the aim of reducing points of transmission (Table 3, M5).

One participant spoke of conducting a mass work from home exercise prior to the national lockdown announcement to test the capacity of IT networks, as it was acknowledged that ‘IT was going to be our biggest risk whether we could get people working remotely’ (Table 3, M6). One organization spoke of taking proactive measures at the start of the lockdown period with regards to priority services customers and the provision of bottled water (Table 3, M7).

With regards to mitigating the threat posed by prolonged high temperatures and large proportions of the population being at home throughout the day, one company spoke of pre‐empting increases in demand and deploying tankers to ‘top up’ the water supply network in advance (Table 3, M8).

### 3.3 Adaptation

Participants actively discussed the measures taken to adapt working practices to reduce the impact of COVID‐19 on operational delivery. Within each response, participants made a clear distinction between office and field‐based staff (Table 4, A1).

**TABLE 4:** Adaptation measures implemented by organizations

Ref	Measure	Qualitative explanation
A1	Office vs. Field staff	‘There were two parts to the way we dealt with it. Probably more than two parts. We've got field staff in operations and we have got office‐based staff … For field‐based staff, the world did change but not in the same way’
A2	Stopping of field‐based operations	‘Our capital works programmes were going on, say our … treatment site. Our staff were nervous about these guys coming onto their site so there was a bit of protecting our own staff … more or less the entire capital works programme was stood down by the end of March’
A3	Use of technology	‘… when we came to commission some of our capital projects … we had some things we needed to get done by the end of the AMP [Asset Management Plan] so we had people using phones to guide us, we had a critical worker self‐isolating … so we were commissioning via WhatsApp … Necessity is the mother of invention, you find a way of doing stuff’
A4	Deployment of upskilled staff	‘There was one treatment works in particular where we lost 50% of the site staff so we did actually deploy a couple of reservists … [who] covered shifts’
A5	Ability to work from home	‘I think there is an acceptance here that we will never be back working in the same way we worked before … The staff survey has shown us that there are about 100 … staff that are keen to get in back to the office, 450–470 [want] … the ability to work from home as well as some time in the office and … [some] who just don't want to go back to the office at all. The vast majority are in the middle … we will never be back to having 100% of people in the office 100% of the time’
A6	Reactive vs planned measures	‘Yeah it largely went to a reactive position and everything was risk assessed to say, do we really need to be doing that activity at the point or can we hold it off? Especially, in the early days we risk assessed each activity then … as the peak of the outbreak began to tail a bit we started looking at them with a risk‐based approach and we started to say can we start to feed them back in? Or do we still need to exercise caution? That is still going on’.
A7	Changes to incident management structures	‘The bronze teams in normal operational activity would have been … geographically split and … operationally focused. Whereas … this time … the bronze teams were functional or directional so we had HR, we had an asset delivery we had a comms [communications] team … completely different to an operational event’

For routine field‐based operations, many activities stopped as a result of the uncertainties regarding the transmission of the virus (Table 4, A2). Customer visits were stopped and planned maintenance activities were scaled back or prioritized. This was to support social distancing and reduce transmission of the virus within personnel. Some organizations staggered their shift patterns and trained staff to conduct remote handovers. A couple of participants explained how critical key workers self‐isolated to ensure that they would be able to continue operational delivery throughout the pandemic. Site visits were also conducted remotely to reduce face‐to‐face contact and innovative approaches were taken to adapt to this new way of working (Table 4, A3). Safety visits were conducted via phone with operators sharing site information via live video and drones. Participants also provided examples of workforce adaptation where staff were identified and ‘upskilled’ to conduct critical roles to cover cases of absenteeism (Table 4, A4; Figure 3a).

For office‐based staff, participants discussed working from home as an ‘overnight digital transformation’ and a move towards a more agile workforce. However, it was the return to the office environment that was discussed within the context of adapting working practices. It was largely perceived that the office environment would adapt as a result of the pandemic and the need to continue social distancing. The ability of many personnel to be able to work effectively from home also demonstrated that a flexible approach could be achieved (Table 4, A5; Figure 3a).

Participants discussed many adaptation measures as reactive rather than planned. This was largely in response to the scale of the pandemic and the rapid timescales with which the country went into a lockdown situation (Table 4, A6). During the initial stages of the pandemic, there was a great reliance on the use of risk assessments to adapt to the immediate situation. However, applying the risk management approach to achieve future resilience created many challenges as a result of uncertainties regarding virus transmission and the possibility of further lockdowns.

Changes to the incident management structures of teams within organizations that were tasked with facilitating a response was also highlighted as an adaptation measure. The traditional operational roles required for operational incidents and events were no longer a focus and were instead replaced by individuals from Human Resources and Communications (Table 4, A7). This was particularly experienced at the operation level of incident management (Bronze) rather than tactical (Silver) or strategic (Gold) levels.

### 3.4 Coping

Participants outlined multiple coping measures and mechanisms. The most prevalent coping measure discussed was that of the move to working from home for a large proportion of office based staff, with many participants reporting the relative success of the move (Table 5, C1).

**TABLE 5:** Coping measures implemented by organisations

Ref	Measure	Qualitative explanation
C1	Working from home	‘You know shifting thousands of people to work from home pretty much over night with hardly any operational impact was really good’ ‘… It's been hard for [people who work from home] to create boundaries with how they work … They don't have a commute, they don't have an effective start and they don't go home at night … a guy on my team said [he] misses the train journey and I said, “you are kidding” and he said, “no I miss it because it was closure for the day”’
C2	Mental health and wellbeing	‘… we were really conscious about people's mental health and the fact that some individuals were now working from home and potentially not engaging with individuals on a day‐to‐day basis. Particularly if you are someone that lives on their own … We are actually really mental health aware but I think we took it to another level with the lockdown’
C3	Communication	‘… the communication protocols we put in place as a business, exec level down to the field teams have been the thing that have given us the ability to cope. There have been lines of communication [and] they have been effective because information has travelled quickly from source to action’ ‘I think in a way the use of Zoom or Teams … [is] less personal because you are on a screen and not physically in a room with someone but what I’ve found is … it actually makes it easier to communicate with large numbers of people … During the lockdown I’ve found I did this weekly for the first few months and I’m doing it fortnightly now … So that's definitely worked well and I think it's changed other practices that we will keep regardless of the restrictions’
C4	Pre‐existing status of remote workers	‘Our field staff are already remotely based … They work out of their van, [where] they have their laptop and they don't have to go into anywhere to be able to log onto anything so … they were relatively safe in coming to work every day, because their office was their van … They would by‐and‐large either be on site on their own or with one other person’

However, the move was not without its challenges with many participants reporting issues and associated disadvantages to the new way of working. The majority centred on employee's wellbeing and mental health because of feelings of isolation or the inability to separate work and home life. Multiple participants therefore spoke of mechanisms implemented at an organizational scale to aid employees ability to cope with regards to their mental health and wellbeing (Table 5, C2).

The use of effective and efficient communication was also considered to be a large part of the organization's ability to cope with the pandemic and new ways of working by participants. As the threat and its associated impacts continued to develop, effective lines of communication put in place across organizations were discussed as coping mechanisms. Increased use of video calling technology was repeatedly spoken about in the context of coping, both with regards to conducting everyday tasks that could no longer be done in person as well as providing a platform for communication that field staff have the ability to access as well as office‐based staff (Table 5, C3).

The pre‐existing status of remote field workers in the water industry was also discussed within the context of coping. Within the water industry, many field workers have the equipment and technology to be based out of their van and therefore do not require access to office spaces (Table 5, C4).

### 3.5 Learning

Participants discussed numerous learnings that have so far occurred from the pandemic, with a view that many more are still to come. The notion of ‘realization of risk’ and the need to *‘*expect the unexpected**’** was mentioned by multiple participants regarding lessons learned (Table 6, L1). As the industry failed to adequately prepare for the scale of the pandemic, one participant suggested the need to now evaluate the organisations risk register in order to test other assumptions they may have made for other potential threats. The failure to see and acknowledge national lockdown and the associated impacts and consequences as a credible scenario that would result from a pandemic threat, meant many companies miscalculated the associated impact.

**TABLE 6:** Learning measures identified by organizations

Ref	Measure	Qualitative explanation
L1	Realization of risk	‘What I'm recommending to the board … is that we really seriously need to look at our risk register and test all our assumptions out again, because if we were slightly wrong about flu pandemic, we weren't expecting lockdown, what else are we slightly wrong about’ ‘[The pandemic] necessarily wasn't classified in terms of impact in the right way. So from that perspective, it wouldn't have been seen as one of the ten or twenty corporate risks … We've not really lived in that type of risk materializing … it's probably caught a few companies off guard in that respect’
L2	Changing view of emergency management	‘Our disaster recovery plan for the head office was if it burnt down you would move to a separate office. So you paid for a disaster recovery office … but clearly now we have just said well actually if it burns down then we just go home. So we stopped that contract’
L3	Success of collaboration	‘No definitely I think that's been really good, even just from a sense check of are you doing the right thing. I think again that was originally set up that format for Brexit but we used that same structure for this and worked really well’ ‘Industry‐level liaison I think has come on in the last few years and we have broken down a few barriers with actually recognizing that there is some strength in numbers and that it's best to share best practice’
L4	Review of traditional working practices and spaces	‘Yeah and other lessons learnt really I think it's given us a great insight into we have been very traditional in the way that we run our business you know desk time and office space is seen as a measure of effectiveness in some ways but we have performed extremely well without all being crammed into a glass box in the middle of [location]’ ‘… we are now looking at reducing the occupancy of our office … to create a better environment. Rather than think oh well we have to come into work, well no you don't have to come in because you can work from home if that suits you … So when you come in you are coming in for a reason to meet your team or do a workshop or work through some idea … So that's definitely something we have learnt really’

As previously outlined, planning done in preparation for the UK’s departure from the European Union was considered a success in regards to preparation. The success of the framework adopted, in order to facilitate such industry‐level collaboration has resulted in the knowledge that such a level of collaboration can benefit the industry as a whole (Table 6, L3).

The relative success of the mass move to working from home has resulted in a changing view of emergency management, with multiple participants discussing ending contracts for backup physical office spaces (Table 6, L2). The mass move to working from home has also provided an opportunity for organizations to redevelop how traditional office spaces are both physically and mentally, viewed, approached and utilized (Table 6, L4). As the traditional view of ‘presenteeism’, and the notion that office‐based employees are at their most productive when sat at a desk in a communal office space has been brought into question by the new way of working. Changes to ways of working have also provided an opportunity to redesign office spaces and to create an environment designed for more specific purposes.

## 4 DISCUSSION

The research has revealed the COVID‐19 pandemic to be a ‘threat multiplier’ (Neal, 2020), as an interaction between the threat of the pandemic and reduced rainfall and higher than average temperatures, have increased the complexity of the system failure modes, impacts and consequences (Figure 2). When applying the Safe and SuRe approach (Butler et al., 2016), COVID‐19 can be classified as an external acute threat because of the fast and unexpected nature of the global progression of the virus. However, pandemic influenza and emergent infectious diseases have both ranked highly on the UK risk register, for many years now (Cabinet Office, 2017), thus pushing it closer to the acute/chronic threat boundary.

Failure of organizations to prepare for, and mitigate the threat was discussed throughout the interviews. Although all had flu pandemic‐based business continuity plans, the plans themselves failed to take into account the scale of the threat faced. With many participants highlighting a national lockdown as a scenario in which they were not prepared for. Multiple participants held the view that the failure of previous pandemic threats to materialize at scale in the UK, resulted in many organizations being caught ‘off guard’. A continued focus on the identification, impact and likelihood of threats at the corporate and organizational level highlights that the sector remains focused on a traditional risk management approach. Such an approach has a tendency to assess the impact and consequence of single threats and hazards. However, as demonstrated within Figure 2, this event consisted of two threats (COVID‐19 pandemic and weather hazards) resulting in a highly complex series of impacts and consequences across social, economic and environmental sectors of society.

Other mitigation‐based interventions such as ‘up‐skilling’ staff to cover operational roles, and the collaborative efforts of the sector regarding the security of supply chain, proved effective at minimizing the scale of the potential effect that the initial wave of the pandemic had on operational performance. Such efforts, which had resulted from learnings from previous industry‐based incidents (Industrial action 2018, Cryptosporidium outbreak 2015, Foot and mouth 2001, 2007), emphasizes the importance of systematic learning for increasing the reliability, resilience and sustainability of systems.

When considering the water system as a social‐ecological‐technical system that comprises of natural, physical, organizational and social systems (Butler et al., 2016), there are multiple potential resulting system failure modes. The cascading effects of system failure modes triggered by the threat of COVID‐19 (Figure 2), and a prolonged period of reduchated rainfall and higher than average temperatures, only further emphasize the complexity of the sector. Thus reinforcing Neal (2020, p. 439) view that COVID‐19 has provided a ‘harsh lesson in complexity’ for water systems. The external functional middle states of national lockdown and a mass move to working from home, resulted in a change to previously predictable patterns of demand and use of the water and wastewater systems (Marshallsay, 2020), as demonstrated within Figure 2.

Such changes to usage patterns and behaviours, resulted in the need for further adaptation and coping mechanisms to be implemented by water companies that had not previously been considered. The mass move to working from home, pushed IT systems to the brink of failure with some requiring more time than others to extend bandwidth and capacity to enable employees to effectively work from home (Figure 2). The global nature of the pandemic meant those relying on overseas service providers had a further reduced ability to adopt ways of working because of the level of dependence on local infrastructure and the quality of technology available. As the working environment extended into people's home and private spaces, the scope for external structural and functional failure modes also increased. Employee's access to broadband and equipment, suitable working space and existing care responsibilities (Cotterill et al., 2020) had the ability to further impact overall organizational performance. Such changes have for the first time altered the traditional dynamics of work and home life, with employers now requiring additional information on employees personal life to maintain organizational performance, as the boundaries between the two continue to blur.

The global requirement to ‘socially distance’ and self‐isolate following contact with a suspected infected individual changed the dynamics of the available workforce in an operational industry, and thus methods for adaptation and coping. Advances in technology have not only provided mechanisms in which operational staff could provide technical knowledge and assistance to on‐site employees without being physically present but have also provided the capability for large organizations to maintain successful and effective lines of communication with their workforce when spread out across large geographic areas.

Existing modes of working such as mobile workers and remote network control were considered crucial to the ability of organizations and specifically operational staff to cope. Increases in organization's efforts to support employee health and wellbeing during this period were also recognized as coping mechanisms, as the ability of the social aspect of the system to negatively impact overall operational and organizational performance is increasingly recognized.

Resulting consequences such as increases in operational expenditure (OPEX), reduction in commercial revenue and increased work–life balance for employees (Figure 2) are shown to be the result of multiple cascading impacts. Figure 2 highlights an imbalance in consequences with more social and economic consequences compared with environment. The reasoning behind this is considered threefold. In many cases, environmental consequences take longer to actualize when compared with social and economic, which are often much more pressing. Water sector regulators across the United Kingdom either completely stopped or largely reduced environmental monitoring and sampling programmes during the first nationwide lockdown, therefore in many cases outside of wastewater site process data, environmental performance data simply does not exist. Finally, the interventions required to address the initial threat posed by the pandemic were predominantly socio‐economic measures which has subsequently resulted in socioeconomic‐based consequences.

The success of industry‐level collaboration and the use of cross‐sector working groups has again highlighted the benefit of collaborative working. It is, however, important to note the context in which the sector operates. Water and wastewater providers in England and Wales are privatized entities that operate within a competitive and highly regulated market. League tables published by industry regulators, the current 5‐year regulatory periods, and the resulting continued race for the top were all cited by some participants as a barrier to past collaboration. Such context therefore has the potential to impact organizations degrees of freedom, and access to resources, if they are to affect true change (Cook & Nemeth, 2010).

The relative success of the move to working from home for a large percentage of the workforce has provided opportunities for organizations to revaluate use of the physical work environment. All participants stated that their organizations working practices would not go back to pre‐pandemic status and would instead focus on a blended working approach, with some organizations already ending contracts for backup office space for use in the case of a site‐based emergency.

## 5 CONCLUSIONS

This study was conducted during the first wave of the COVID‐19 pandemic in the United Kingdom, with the final interviews conducted as the UK re‐entered a phase of lockdown. Semi‐structured interview questions were designed around the four interventions outlined in the Safe and SuRe framework. Findings following the analysis of the sectors’ organizational response to the COVID‐19 pandemic are also presented using the Safe and SuRe framework. The key conclusions drawn from this research are as follows:

1. COVID‐19 was found to be a ‘threat multiplier’. Interaction with the weather hazards of reduced rainfall and higher than average temperatures increased the complexity of resulting cascading effects and provided organizations with an insight into the complexity of an acute threat. The reliability, sustainability and resilience of organizations, systems and networks have therefore been tested with many lessons learnt.
2. The pandemic highlighted pre‐existing system vulnerabilities, with a realization of risk noted across the industry, as repeated modes of failure, impacts and consequences, independent of causal threat, were emphasized.
3. Cross‐industry preparation, collaboration and collective working were found to be successful and effective by all organizations involved. With many expressing interests in actively pursuing such options for the future and a need to emphasize the benefits of such modes of working to industry regulators.
4. Traditional working practices were altered, and the use of technology was central to required adaptation and coping mechanisms. The pandemic also provided the opportunity for organizations to re‐evaluate traditional physical working spaces and environments.
5. Pre‐existing pandemic plans were inconsiderate of the scale of the COVID‐19 pandemic, resulting in some organizations now re‐evaluating other existing business continuity and response plans. One participant even reflected that wider risk management approaches warranted an overhaul. Although the overall view remains that water companies did well to respond to the pandemic and maintain the performance of critical services, it is important that organizations do not become complacent and must fully acknowledge and embed new knowledge in best practises.

## ETHICAL APPROVAL

Ethical approval was sought and received from the College of Engineering Maths and Physical Sciences (CEMPS) Research Ethics Committee, University of Exeter on 18.05.20 (Ref: eEMPS000265 v3.0).

## Data Availability

The data that support the findings of this study are available on request from the corresponding author. The data are not publicly available due to privacy or ethical restrictions.
